# Closed-loop control of zebrafish behaviour in three dimensions using a robotic stimulus

**DOI:** 10.1038/s41598-017-19083-2

**Published:** 2018-01-12

**Authors:** Changsu Kim, Tommaso Ruberto, Paul Phamduy, Maurizio Porfiri

**Affiliations:** Department of Mechanical and Aerospace Engineering, New York University Tandon School of Engineering, Brooklyn, New York, 11201 USA

## Abstract

Robotics is continuously being integrated in animal behaviour studies to create customizable, controllable, and repeatable stimuli. However, few systems have capitalized on recent breakthroughs in computer vision and real-time control to enable a two-way interaction between the animal and the robot. Here, we present a “closed-loop control” system to investigate the behaviour of zebrafish, a popular animal model in preclinical studies. The system allows for actuating a biologically-inspired 3D-printed replica in a 3D workspace, in response to the behaviour of a zebrafish. We demonstrate the role of closed-loop control in modulating the response of zebrafish, across a range of behavioural and information-theoretic measures. Our results suggest that closed-loop control could enhance the degree of biomimicry of the replica, by increasing the attraction of live subjects and their interaction with the stimulus. Interactive experiments hold promise to advance our understanding of zebrafish, offering new means for high throughput behavioural phenotyping.

## Introduction

Robotics is changing the way we progress as a society, by transforming our pathways to scientific inquiry and discovery^1^. The field of “ethorobotics” is a striking example of how robotics can empower scientists with unprecedented tools to advance our understanding of animal behaviour in the laboratory and in the field^2–7^. The application of robots to elicit a target behavioural response in animals could be grounded upon the fundamental ethological concept of “social releasers”, which constitute the influential cues that trigger animal interactions^8^. The last decade, in particular, has witnessed a surge in the use of robots to elicit standardized, repeatable, and consistent response in live subjects across a wide array of behavioural studies, seeking to understand emotions, perception, and cognition^9–12^. In these experiments, robots are engineered to display desired morphological features and perform complex locomotory patterns, drawing inspiration from live animals, often with a high degree of biomimicry.

While we have seen incredible progress in animal-robot experiments, most of the studies have been performed in “open-loop control”, where the robot is pre-programmed to implement a-priori-chosen behaviours, without being responsive to the behaviour of the live subject. In this sense, the robotic stimulus does not actively interact with the live subject, which is instead influenced by the presence and movement of the robot. Such open-loop controlled robots have been successfully integrated in the study of animal behaviour across a wide range of taxa, from insects to fish, birds, and mammals^7,13–30^.

Robotic models have been utilized to examine the role of visual and chemical cues involved in the honeybee (*Apis mellifera*) dance communication system^13,14^. Several studies on birds have been made possible using robotics, ranging from research on the courtship of male satin bowerbirds (*Ptilonorhynchus violaceus*)^15^, to the analysis of attachment bonds and spatial exploration in chicks of quails (*Coturnix coturnix japonica*)^16,17^. Robotic fish have been implemented in several behavioural assays, on social fish species including zebrafish (*Danio rerio*)^7^, bluefin killifish (*Lucania goodei*)^18^, giant danios (*Devario aequipinnatus*)^19^, golden shiners (*Notemigonus crysoleucas*)^20–22^, trunkfish (*Mormyrus rume*)^23^, mosquitofish (*Gambusia affinis*)^24^, mackerels (*Scomber scombrus*)^25^, Siamese fighting fish (*Betta splendens*)^26^ three-spined sticklebacks (*Gasterosteus aculeatus*)^27,28^. These studies have helped answer a number of important questions, from the effect of hydrodynamic advantage in schooling to the role of morphophysiological factors on courtship and aggression. Robotic rats imitating the physical motions of living rats, such as body grooming and rearing, have been used to investigate the social behaviour of rats (*Rattus norvegicus*)^29^, and multisensory communication of squirrels (*Sciurus carolinensis*) has been investigated through robots displaying alarm behaviour in a field study^30^.

Although these robotic tools have helped in supporting hypothesis-driven studies on animal behaviour through customizable and versatile stimuli, they did not afford the dynamic interplay that is distinctive to animal interactions. To facilitate the integration of robotic stimuli in animal experiments, a number of studies have investigated “closed-loop control” systems, in which robotic stimuli could adapt to the behaviour of fish^31–35^, birds^36–38^, insects^39–41^, and mammals^42,43^. In an authentic closed-loop control system, the robotic stimulus interacts in real-time with live subjects, based on automatic scoring of animal behaviour^6^. For example, a robotic replica was actuated based on the position of a school of golden shiners using a wheeled support magnetically connected to the robot^31^, and a similar strategy has been recently proposed for zebrafish^35^.

In some of these closed-loop control systems, computer vision was utilized to measure animal behaviour in real-time and sustain a feedback loop between animals and robots. For instance, computer vision techniques were used to modulate the tail-beat frequency of a robotic fish with respect to the position of a zebrafish^32^; track the centre of a shoal of guppy fish (*Poecilia reticulata*) to offer feedback to a replica actuated by a mobile robot^34^; and acquire the position of a school of golden shiner to create feedback for a robotic predator^31^.

In this study, we propose a first, interactive robotics-based platform to study the behaviour of zebrafish (*Danio rerio*, Hamilton, order Cypriniformes, family Cyprinidae) in three dimensions (3D). Zebrafish, our target species, is a freshwater fish which is recently emerging as a species of choice for hypothesis-driven studies in several research fields, such as developmental genetics^44^, translational neuroscience^45^, and toxicology^46^. This animal model presents several unique features, such as a high level of sociality^45,47^, elevated number of genetic and neural homologies with humans^48,49^, and ease of husbandry and maintenance^50^. Zebrafish has a uniquely complex repertoire of 3D swimming patterns^51^, including fast horizontal and vertical movements, in a so-called burst-and-coast style^52^. Over the years, we have demonstrated the feasibility of using robotic stimuli in zebrafish research to study the determinants of social behaviour^7,53–55^ and fear response^56,57^, along with the effect of pharmacological manipulations^57–59^. The potential of robotics to support zebrafish research in the study of complex brain disorders has already been acknowledged by a number of researchers^60–62^.

The platform is grounded in recent advancements by our group on the design of biologically-inspired robotic stimuli for the study of fish behaviour in open-loop experiments^53,54^. We integrate 3D printing and real-time computer vision tracking to establish a closed-loop control system, in which a 3D-printed replica of a live fish is manoeuvred in 3D by a robotic arm, based on real-time measurements of fish position. The platform allows for actuating the replica through four independent degrees of freedom, including 3D translational movements and single axis rotational motion to proxy fish tail-beating.

In contrast with our previous efforts^53,54^ where predetermined trajectories were implemented based on independent observations of live fish motion, a custom-made tracking software was developed to allow for real-time tracking of the position of the focal subjects in the experimental tank. Differently from existing studies using 2D position tracking of focal subject to sustain closed-loop control^31,32,34^, this system affords, for the first time, real-time tracking and closed-loop control in 3D. The effectiveness of the new robotic platform was tested on zebrafish in a set of binary choice experiments, in which fish were systematically presented to a biologically-inspired replica with different levels of interactivity (ranging from full open-loop to closed-loop control in 3D). While the platform enables the implementation of a wide range of control schemes, we focused on a feedback loop in which the replica would seek to mirror the behaviour of the focal subject, by following some of its movements.

We hypothesized that the behavioural response of the focal subject would vary as a function of the degree of the interactivity of the replica. Specifically, we predicted an increased preference, measured as time spent close to the replica, for closed-loop controlled systems, where the replica would respond to the focal subject and mirror its behaviour. This increased preference was expected to be associated with a higher degree of biological mimicry, thereby accompanied by the absence of any stress-related responses, measured in the form of freezing or geotaxis^52,63^.

To detail the interaction between the replicas and live fish, we applied the information-theoretic framework of transfer entropy^64^. Transfer entropy measures the information flow between two coupled dynamical systems from their raw time series, offering a quantitative insight into potential cause-and-effect relationships between them. This approach has been demonstrated in several other efforts to evaluate study cause-and-effect relationships between animals and robots^65^, leadership-followership relations in pairs of bats^66^, and predator-prey interactions^67^, offering a model-free data-driven perspective on behavioural interactions.

Within this information-theoretic approach, we predicted that transfer entropy would help in detecting the cause-and-effect relationships underlying the behaviour of the replica and quantifying the role of the feedback from the replica. Specifically, we expected that transfer entropy would detect a net information flow from the replica to the focal subject in open-loop control, where the replica was unresponsive to the motion of the live fish. Instead, in closed-loop control, we anticipated a net information flow from the focal subject to the replica, whose movement was actuated in real-time to follow the live fish. Finally, we predicted that closed-loop control would create a positive feedback mechanism in the behaviour of the live subject, leading to an increased information flow from the live fish to the replica, and vice versa.

## Materials and Methods

### Ethics statements

Experiments were performed in accordance with relevant guidelines and regulations, and were approved by the University Animal Welfare Committee (UAWC) of New York University under protocol number 13-1424.

### Animals and housing

A total of 70 adult wild-type zebrafish were used in the experiment. The fish were purchased from an online aquarium retailer (LiveAquaria.com, Rhinelander, WI, USA), with a one to one nominal ratio in males and females. Fish measured approximately 3 cm in body length (BL) at the beginning of the experiment. They were housed in 37.8 l (10 gallon) water tanks for an acclimatization period of 15 days prior to the experiment, with a maximum density of 20 fish per tank. Water parameters were regularly checked, and temperature and pH were maintained at 26 °C and 7.2 pH, respectively. Animals were kept under a 12 h light/12 h dark photoperiod^68^ and fed once a day around 7 pm with commercial flake food (Hagen Corp. Nutrafin max, Mansfield, MA, USA). After acclimatization, a total of 10 fish were randomly selected as live stimuli, and separated from the others in a 10-gallon tank. The remaining 60 fish were used as focal subjects for the 60 trials comprising our study.

### Apparatus

**Figure 1:**
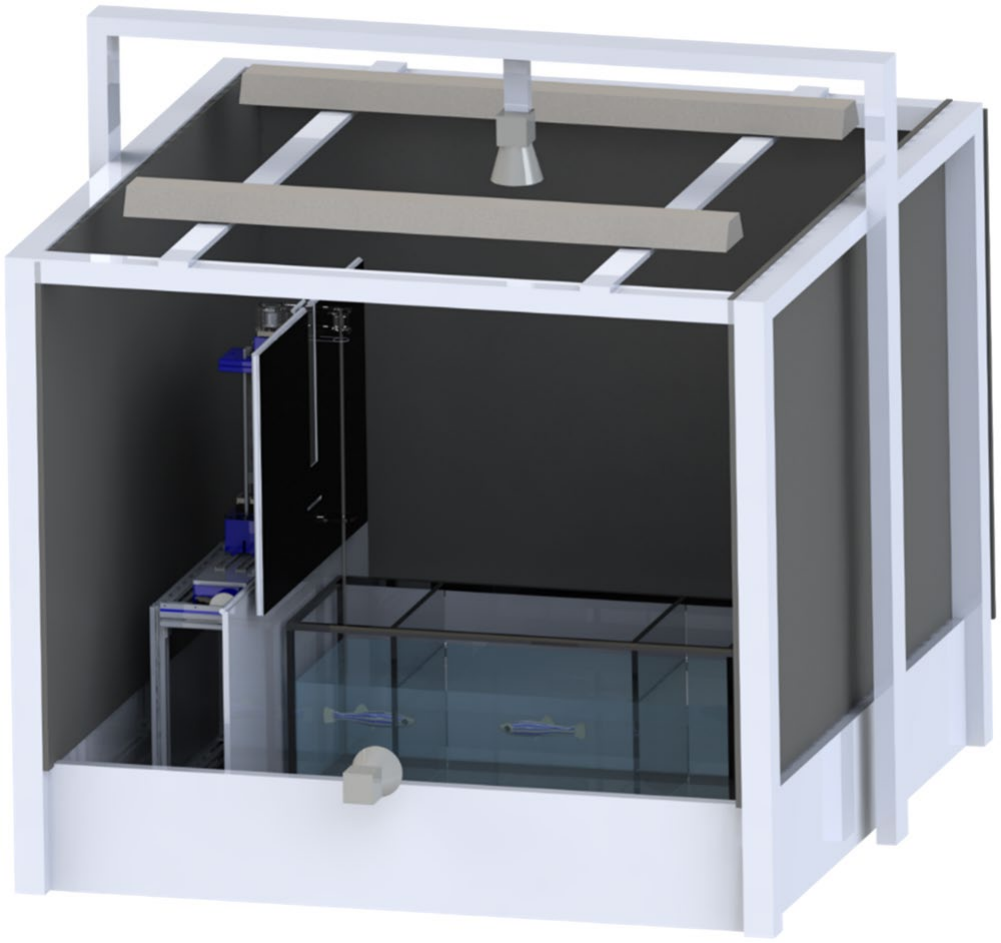
Sketch of the experimental apparatus. The drawing shows the experimental tank, robotic platform, lightings, cameras, and holding frame. For clarity, the black curtain on the front of the frame is omitted and the focal fish and the robotic stimulus are magnified.

The experimental apparatus consisted of a glass water tank measuring 74 × 30 × 30 cm (length, width, and height), partitioned into three compartments using transparent acrylic panels (McMaster Corp., Waltham, MA, USA), as shown in Fig. 1. The two lateral compartments measured 10 × 30 × 30 cm (length, width, and height), and the water level was kept at 15 cm. The tank was placed within a holding frame of dimensions 120 × 120 × 20 cm (length, width, and height), made of aluminum T-slotted bars and acrylic plates.

Two 25 W white fluorescent lights (All-glass aquarium, Franklin, WI, USA) were mounted at 78 cm from the water surface and orientated toward the tank to illuminate the apparatus. Two cameras (Logitech C920 webcam, Lausanne, Switzerland) were used to record videos of the focal fish and the stimulus at 30 frames per second with a resolution of 640 × 480 pixels. One camera was placed at the same height of the lights, pointing at the center of the water tank, to capture the horizontal motion of the zebrafish and the stimulus. The other camera was mounted on a tripod at a distance of 58 cm in front of the water tank and centred at the water level to capture their vertical motion.

The bottom walls of the tank and holding frame, the two short side walls of the tank, the lateral walls of the holding frame were all covered with white contact paper. To ease the tracking of the fish and stimulus from the frontal view, a sheet of white contact paper was used as background. This sheet was placed 25 cm away from the tank to reduce asymmetries in the perception of the environment by live fish. Black curtains surrounded the experimental apparatus to reduce external disturbance.

### Robotic platform

**Figure 2:**
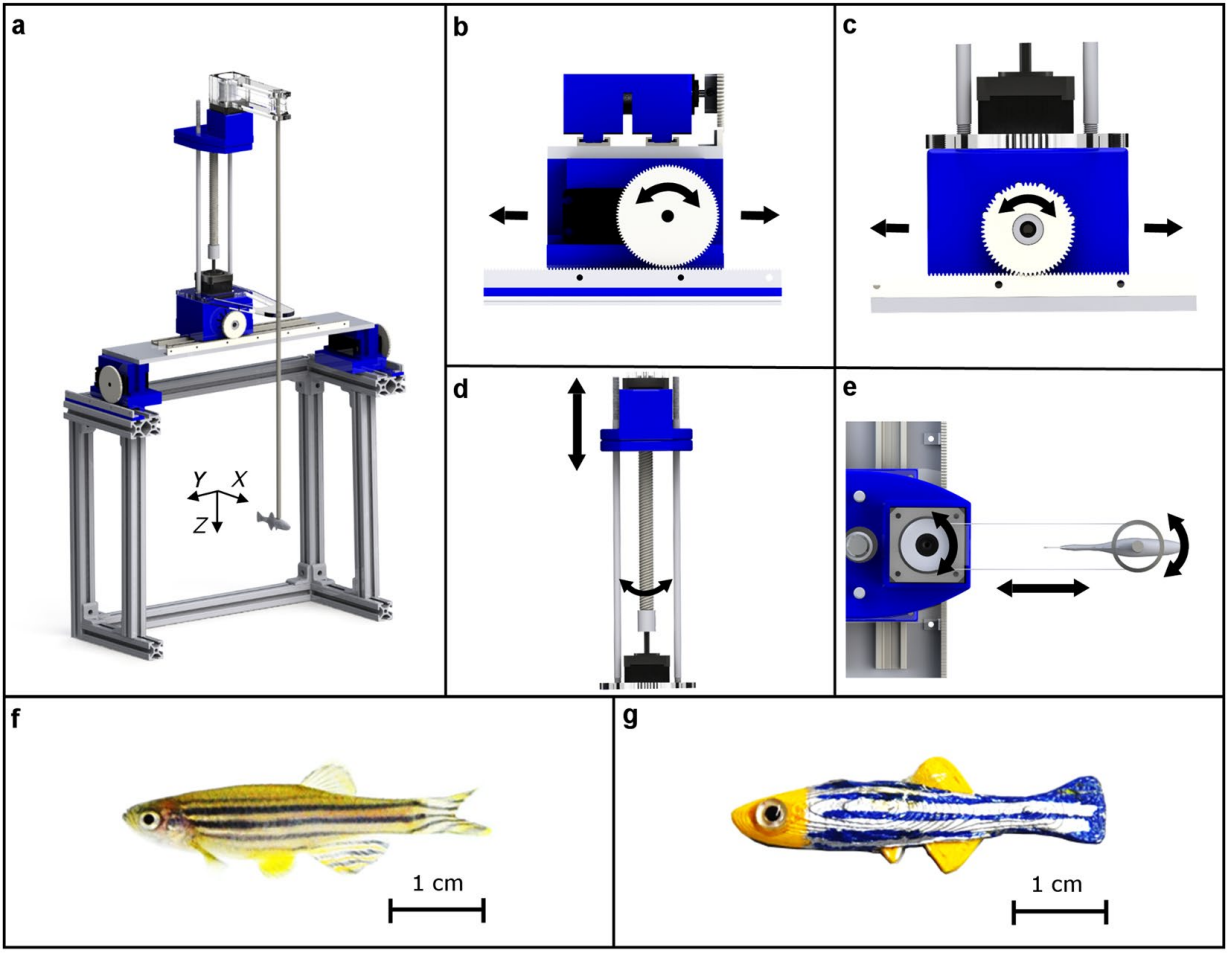
Elements of the robotics-based experimental approach. This composite picture presents: a general overview of the platform used for manoeuvring a 3D-printed zebrafish replica (**a**); detailed movements of the platform along the *X*-axis (**b**), *Y*-axis (**c**), and *Z*-axis (**d**); rotation of the replica (**e**); a live zebrafish (**f**); and biologically inspired 3D-printed replica of the zebrafish (**g**).

An interactive 3D robotic platform (Fig. 2a) was designed to control the motion of a 3D-printed zebrafish replica. Similar to our previous work^53^, the platform was designed to actuate the replica in 3D, and execute small body oscillations at a frequency of 2 Hz, proxying the tail beating motion of a live fish. The platform frame was constructed from aluminium T-slotted bars (McMaster Corp., Waltham, MA, USA) and 3D-printed parts. The 3D-printed parts were designed using the computer-aided design software SolidWorks (Dassault Systems SolidWorks Corp., Waltham, MA, USA) and fabricated with a Dimension Elite 3D printer (Stratasys, Eden Prairie, MN, USA) in acrylonitrile butadiene styrene (ABS) material.

Two servo motors (HS-755HB, Hitec RCD, Poway, CA, USA), located at the top of the platform, were connected to a rack-and-pinions gear (Robotzone, Winfield, KS, USA) and used to actuate the platform along the *X*-axis, as shown in Fig. 2b. A DC motor with an encoder (Robotzone, Winfield, Kansas, USA), mounted to a rack-and-pinions gear (Robotzone, Winfield, KS, USA), was used to control the movement of the replica along the *Y*-axis (Fig. 2c). A stepper motor (NEMA-17, Adafruit, New York, NY, USA) was utilized to actuate the vertical motion of the replica by using a threaded rod (McMaster Carr, Elmhurst, Illinois, USA), which transformed rotational motion into translational movement along the *Z*-axis (Fig. 2d). Another stepper motor (NEMA-17, Adafruit, New York, NY, USA) was used to actuate the oscillations of the replica (Fig. 2e), and it was mounted on a cantilever traveling along the threaded rod.

Following our previous work^53^, the morphology and colour pattern of live zebrafish (Fig. 2f) served as the inspiration for the design of the replica (Fig. 2g). Specifically, the replica was designed in SolidWorks, and 3D-printed in yellow ABS thermoplastic using the Dimension Elite 3D. The replica measured 3 cm in length, and it was painted with non-toxic colours (Krylon, Krylon Products Group, Cleveland, OH, USA) to mimic the blue and silver striped pattern of live zebrafish. In addition, glass eyes (Van Dyke Supply Co., Granite Quarry, NC, USA) were attached to the replica to accurately mimic fish morphology.

The replica was connected to a 6.35 mm diameter acrylic rod (McMaster Carr, Elmhurst, IL, USA), which was fixed to a 24 mm diameter pulley printed in a transparent material (Objet Vero clear RGD 810, Stratasys, Eden Prairie, MN, USA) using a dedicated 3D printer (Connex500, Stratasys, Eden Prairie, MN, USA). Another pulley with the same diameter was connected to the stepper motor employed to elicit the oscillating motion. Two acrylic plates connected to the cantilever were used to hold the two pulleys. The two pulleys were coupled with a clear nylon line (McMaster Carr, Elmhurst, IL, USA), such that the rotational motion of the stepper motor could be transmitted to the rod. The nylon line was tightly coiled around both pulleys to prevent slippage. The platform was concealed using a black fabric curtain and white contact paper to match the colours of the background. The curtain was fixed to the platform using an aluminium support, and two acrylic rods were attached to the side of the experimental tank to hold the contact paper, as shown in Fig. 1.

The motion of the robotic platform was controlled by master and slave microcontrollers (Arduino Uno, Arduino, Italy), an Ethernet shield (Arduino, Italy), and two motor shields (Adafruit, New York, NY, USA). The two microcontrollers were required for simultaneously actuating the two stepper motors. The master microcontroller was utilized to: (i) receive the command for the desired position from the computer; (ii) control the position of the two servo motors (*X*-axis); (iii) adjust the voltage delivered to the DC motor with positional feedback from a magnetic rotary encoder (*Y*-axis); and (iv) regulate the position of the stepper motor regulating the oscillating motion of the replica. The slave microcontroller was commanded by the master microcontroller through serial communication to actuate the position of the stepper motor for its vertical motion (*Z*-axis).

The platform wirelessly received a command, every 0.5 s, via user datagram protocol (UDP) to actuate the motors based on real-time feedback from the 3D position of the focal fish, when closed-loop control was implemented, or on a pre-programmed trajectory. The workspace of the robotic platform, defined as the intended operational spatial limits, was smaller than the stimulus compartment of about 1 BL from each boundary, to avoid collisions of the replica on the walls. Technical details on the control of the robotic platform are available in the supplementary material.

### Real-time tracking software

**Figure 3:**
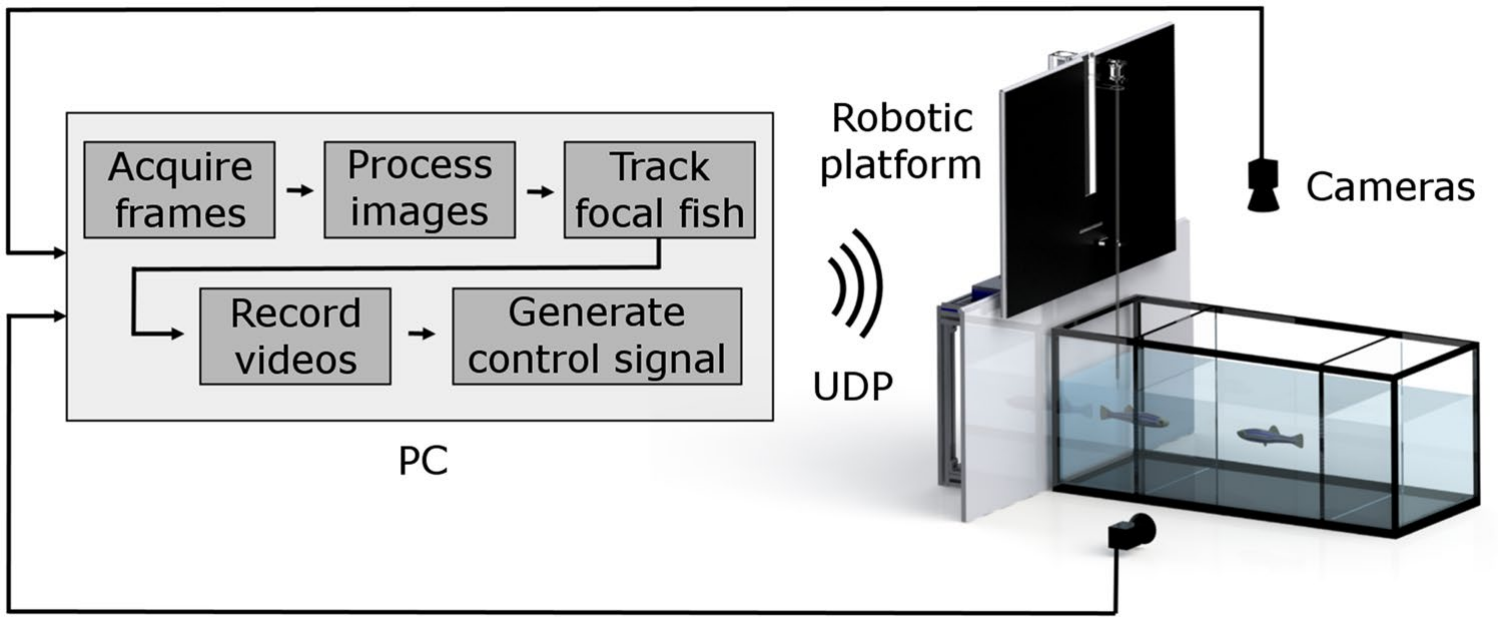
Schematics of the interactive robotic platform. The computerized base station for control feedback received an input (images) from the top and frontal camera. The images were processed and the fish was tracked in real-time to generate a feedback control signal that actuated the robotic platform.

A computer vision-based real-time tracking software was developed using Visual Studio 2015 (Microsoft, Redmond, WA, USA). The software was designed to automatically track the 3D position of the focal zebrafish, and follow it throughout the trial at the full acquisition rate of 30 frames per second, while interactively matching the motion of the replica with the trajectory of the fish. The software was written in C++ language, and was based on the open source computer vision library OpenCV v3.1^69^ (Intel Corp., Santa Clara, CA, USA). The software was programmed to simultaneously activate the two orthogonal cameras (Fig. 3) and read frames for the real-time tracking.

**Figure 4:**
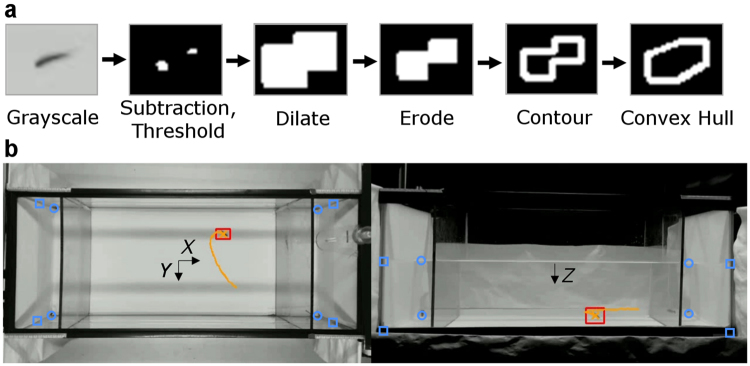
Real-time tracking software process. The picture shows the series of computer vision filters for acquiring the target (**a**), and a sample image of zebrafish tracking in the (**b**) top (left) and front (right) views. The fish trajectory corresponding to 100 consecutive frames is shown in yellow. Corners of the near and far side of the water tank are marked with green squares and circles, respectively.

The zebrafish tracking software was built on a blob (binary large object) detection algorithm^69^ and a series of computer vision filters to robustly detect the location of the focal fish or replica (Fig. 4a). Specifically, for each camera view, the acquired image frame was first converted into grayscale and then blurred with an averaging window of 7 × 7 pixels (corresponding to approximately 8 mm). Successive frames were then subtracted to identify potentially moving targets, observed as differences between the frames. To reduce noise from the image subtraction, a binary filter was utilized on the frame, with a threshold of 8 and 12 (on a 255 grayscale range) for the front and top views, respectively. These thresholds accounted for differences in the brightness and contrast from the two views. Pixels with intensities below the threshold indicated that there was no fish motion detected and were thus treated as part of the background, while pixels with intensities above the threshold were associated with a potentially moving target.

Given the relatively large acquisition frequency compared with the typical speed of zebrafish, the difference between two frames typically resulted in the target being split into two disconnected regions for the head and tail (see Fig. 4a). To create a connected body, a dilation filter was applied, thereby increasing the size of each detected pixel into a 15 × 15 pixel square. A 7 × 7 pixel square eroding filter was then applied to resize the artificially dilated region. Next, to facilitate the identification of target shapes prior to the application of the blob detection algorithm, the software identified the contour of each object and generated a convex hull shape for it. A blob detection algorithm was then applied to locate the centre position, size, and aspect ratio of each object (blob) in the frame.

Although unintended blobs appeared intermittently (such as from surface ripples or reflections of the fish), a single blob of the target was identified in the majority of the images, and this was used to track the position of the target (focal fish or replica). To tackle the problem of unintended blobs and reliably track the target, we implemented three additional methods after 100 frames (or 3.3 s) from the initialization. First, when the tracking of the target was lost due to abnormal behaviour or freezing of the fish, the tracking software searched for other possible blobs within a circular region with a radius of 30 pixels. Second, if the target was not found within the circle, the radius of the search region was expanded by 1.5 pixels for successive frames, until the target was retrieved (and the radius was reset to 30 pixels). Meanwhile, a Kalman filter^70^ was implemented to anticipate the position of the target from the previously tracked positions. Finally, in the event that multiple blobs were detected in the search region, the new target was selected as the blob with the minimum distance from the last tracked position. During tracking, the system automatically marked a success for each frame if a blob was identified. The average success rate of the tracking software over a trial was 91.8% and 84.8% for the front and top views, respectively.

To compensate for the distortion associated with the perspective view from each camera, the 2D coordinates from the front and top views (Fig. 4b) were linearly interpolated based on known physical measurements of the water tank and water column and their 2D inferences^71^. Further details on the interpolation process are available in the supplementary material.

### Experimental conditions

After the interpolation process to obtain the 3D coordinates of the focal fish, the motors of the robotic platform were independently actuated on the *X*-, *Y*-, and *Z*-axes, in either an open-loop (OL) or closed-loop (CL) control (see Supplementary Videos S1 and S2, respectively), to manoeuvre the replica to a desired position within the 3D space. We implemented independent OL or CL control on each axis. In OL control, the robotic platform was actuated in a predetermined trajectory based on observations of a live stimulus fish, without any feedback from the focal fish. In CL control, feedback from the focal fish was utilized in the form of real-time spatial information.

To acquire realistic trajectories for use in OL control, we conducted experiments on the interaction between a live stimulus, placed in a lateral compartment, and the focal fish in the centre (see Supplementary Video S3). Not only was this condition (2-Fish) used for informing the control design, but also it served as a baseline on which to test the efficacy of the robotic stimulus on the focal subject. To mitigate potential confounds associated with the robotic platform, we sought to create an equivalent acoustic signature and visual background to those generated by the motion of the robotic platform. Thus, the robotic platform was placed in the same lateral compartment of the live stimulus and actuated along a 3D trajectory utilized in our previous work^53^. To avoid physical contact with the live stimulus, a shorter rod, without the replica, was used. The swimming pattern of the stimulus fish was tracked and used as the basis for predetermined trajectories in OL control.

In OL control, the robotic platform actuated the replica in the predetermined trajectories acquired from condition 2-Fish. Based on the camera frame rate and the rate of commands sent to the platform, the trajectory of the stimulus fish was downsampled for use in OL control. Specifically, since the observation of the stimulus fish was recorded at 30 frames per second, and position data was sent twice per second, we downsampled the data by a factor of 15. The actuation along each axis was individually implemented to afford the composition between OL and CL controls, such that the motion along an axis could be from the 2-Fish condition while closed loop would be created on another axis.

In CL control, the position of the focal fish, obtained through the real-time tracking software, was utilized as feedback for actuating the replica to follow the focal subject. Specifically, in CL control, we sought to minimize the distance between the focal fish and the replica, with respect to any of the *X*-, *Y*-, and *Z*-axes. Minimizing the distance along the *X*-axis caused the CL control to position the replica to the limit of the workspace, in front of the transparent partition. With respect to either the *Y*- or *Z*-axis, instead, the distance between the focal fish and the replica was minimized by dynamically actuating the replica toward the *Y*- or *Z*- coordinate of the focal fish, respectively. The signal to the motor was generated so that the position of the replica was a scaled down version of the focal fish with respect to the *Y*-axis. Specifically, the geometric centre was placed in the middle of the width of the compartment and the desired position was linearly scaled to match the workspace of the platform. Closing the loop in the *Z*-axis was analogous to the *Y*-axis closed-loop, with a different scaling factor.

**Figure 5:**
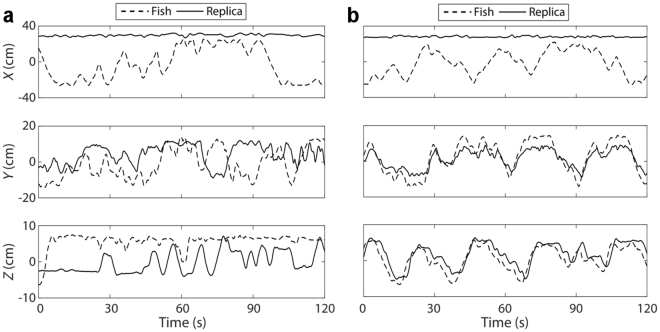
Sample trajectories of focal fish and replica. The graphs illustrate two-minute sample trajectory of the focal fish and the replica along each of the axes in XYZ-OL condition (**a**, open-loop) and XYZ-CL condition (**b**, closed-loop). The trajectory of the focal fish is shown as a dashed-grey curve, and the trajectory of the replica is depicted as a solid-black curve. For *X*, *Y*, and *Z*, respectively, the root mean square errors computed over the entire trial are: 34.85, 11.29, and 6.5 cm (**a**); and 34.78, 4.72, and 3.36 cm (**b**).

While the motion along the *Z*-axis was controlled through a stepper motor that enabled fine position control, an overshoot on the order of 1 body length could be generated by the DC motor for the *Y*-axis control. This overshoot was estimated from the target refresh rate of 0.5 s and the maximum speed of 10.4 cm/s from technical specifications of the DC motor. Typical results of the focal fish and replica positions in OL and CL controls in the *X*-, *Y*-, and *Z*-axes are illustrated in (Fig. 5a and b). Sporadically, slippage between the *Y*-axis motor and its holding case of about 1 mm (clockwise or counter-clockwise) occurred during the trial with a frequency of few events per minute. Finally, the translation along the *Y*-axis might have caused the replica to potentially move backwards few times per trial, for short bouts of approximately one second in duration.

The OL and CL control strategies were utilized to generate five conditions, namely, full open-loop in all three axes (XYZ-OL), closed-loop along the *X*-axis (X-CL), closed-loop along the *Y*-axis (Y-CL), closed-loop along the *Z*-axis (Z-CL), and closed-loop along all three axes (XYZ-CL). In the XYZ-OL condition, the replica was controlled in open-loop long all three axes. We refer to these five conditions as “replica” conditions, in contrast with condition 2-Fish that used a live stimulus.

Specifically, in the X-CL condition, the replica was actuated in CL control along the *X*-axis while the motion along the *Y* and *Z*-axes was controlled in OL. Similarly, in the Y-CL and Z-CL conditions, the replica was actuated in CL along the *Y-* and *Z*-axes, respectively, while the movements along the two remaining axes were controlled in OL. In the XYZ-CL condition, the replica was actuated in CL along all three axes. The control strategies for the five replica conditions are shown in Table 1.

**Table 1:** Control strategies for the robotic platform.

Condition	*X*-axis	*Y*-axis	*Z*-axis
XYZ-OL	OL	OL	OL
X-CL	CL	OL	OL
Y-CL	OL	CL	OL
Z-CL	OL	OL	CL
XYZ-CL	CL	CL	CL

The table shows the control strategies implemented in each replica condition for actuating the replica along the *X*-, *Y*-, and *Z*-axis.

### Experimental procedure

The experiment was conducted from November to December 2016. Immediately after transferring the focal fish to the central compartment of the experimental tank using a hand net, the real-time tracking software was started, and the robotic platform was actuated. Video of the trials were recorded using the real-time tracking software for 20 min, which includes 10 min for habituation and 10 min for observation. Each focal fish and live stimulus was used only once.

Up to ten trials were performed per day, for a maximum of five trials in the morning from 9:00 to 1:30 PM, and five in the afternoon from 2:00 to 6:30 PM. Each condition consisted of 10 trials, in which the stimulus alternated between the left and right lateral compartments between trials. Further, the order of the replica conditions was randomized with an even distribution of trials in the left and right lateral compartments. In each trial, the replica was initially positioned in the lower-left midpoint of the respective lateral compartment.

The 2-Fish condition was conducted before the replica conditions. The trajectory of the live stimulus was acquired from the stimulus of the 2-Fish condition to generate predetermined trajectories. The order of the trajectories used in any of the replica conditions implementing OL control (XYZ-OL, X-CL, Y-CL, and Z-CL) was identical to the 2-Fish condition. That is, the first trial conducted for XYZ-OL, X-CL, Y-CL, Z-CL conditions utilized the predetermined trajectory generated from the first trial of the 2-Fish, and so on.

### Data analysis

Prior to data analysis, the trajectories of the fish and replica were repaired with an *ad hoc* script to correct for tracking errors in the trials. Specifically, errors were identified by examining when the speed of the focal fish or the replica was higher than a threshold value. The threshold value for focal fish speed was set at approximately three times its average speed (20 cm/s in all axes). Similarly, the threshold value of the replica speed was chosen based on estimated maximum speeds calculated from technical specs (*X*-axis: 5 cm/s, *Y*-axis: 13 cm/s, and *Z*-axis: 6 cm/s).

In the event of an error, the *ad hoc* script was allowed to repair the trajectories of a target (either the focal fish or the replica) by facilitating its manual tracking. Specifically, the script slowed down the playback of the trial frames, down to 10 frames per second, such that the experimenter could manually control the mouse pointer to hover it over the target in the frame and replace the erroneous coordinates. After correcting the errors, the trajectories of the focal fish and the stimuli were filtered using the Daubechies-wavelet filter^72^ to reduce the noise and smoothen the trajectories (Fig. 5a and b).

We investigated the behavioural response of the focal fish, including spatial preference, shoaling tendency, and activity. With respect to the spatial preference of the focal fish, we computed the time budgeting along the water column by virtually dividing the water column into two equal partitions of 7.5 cm and counting the time the fish spent in the top and the bottom of the tank. Shoaling tendency was evaluated as the time spent by the fish within four body lengths from the stimulus^73^. With respect to the activity of the focal fish, we evaluated the time spent freezing, computed as the percentage of time in which the fish was not moving in a circle of 2 cm in radius during 2 s^7,32^. Further analysis on the activity of the focal fish and of the stimuli is available in the supplementary material.

Transfer entropy^64^, an information theoretic quantity to infer causal relationships between processes, was used for quantifying the interactions between the fish and the stimulus. Let *Q* and *R* be two discrete stationary stochastic processes, transfer entropy from *R* to *Q* is defined as

1 $${{\rm{T}}{\rm{E}}}_{R\to Q}=\sum _{{q}_{t+1},{q}_{t},{r}_{t}}p({q}_{{\rm{t}}+1},\,{q}_{t},\,{r}_{t}){{\rm{l}}{\rm{o}}{\rm{g}}}_{2}\frac{p({q}_{{\rm{t}}+1}|{q}_{t},\,{r}_{t})}{p({q}_{{\rm{t}}+1}|{q}_{t})},$$

where *q*_*t*_ and *q*_*t+*1_ denote the values of *Q* at time *t* and at time *t* + 1, respectively; *r*_*t*_ and r_*t*+1_ the values of *R* at time *t* and time *t* + 1; *p*(*q*_*t+*1_|*q*_*t*_, *r*_*t*_) is the conditional probability of *Q* at *t* + 1, given its previous state and that of *R*; and Σ represents summation over all possible realizations *q*_*t*_, *q*_*t+*1_, and *r*_*t*_. Transfer entropy is a non-symmetric quantity that measures the reduction in the uncertainty of *Q* given *R*. Net transfer entropy, measured as the difference between transfer entropy from *R* to *Q* and transfer entropy from *Q* to *R*, quantifies the extent of asymmetry in the information flow between *Q* and *R*.

As demonstrated in our previous work^65^, this quantity could be used to infer cause-and-effect relationships in behaviour, such that a positive value of net transfer entropy could be interpreted as *Q* influencing *R*, and vice versa, a negative value of net transfer entropy would identify an influence of *R* on *Q*. We computed transfer entropy between the focal fish and the stimulus in each trial for all experimental conditions using the raw positions along the *Y*- and *Z*-axis. The position in the *Y*-axis was discretized into 10 bins, such that a bin would approximately match 1 BL. The position in the *Z*-axis was discretized into 5 bins, to match the bin size along the *Y*-axis. We evaluated net transfer entropy as the difference between the transfer entropy from the fish to the stimulus and that from the stimulus to the fish. Transfer entropy was computed using Process_Network_v1.4^74^ software.

For each of the experimental conditions, a one-tail one-sample *t*-test with reference mean at 50% was used to assess whether the fraction of time spent by the focal fish at the bottom of the tank was higher than chance. A one-tail one-sample *t-*test with reference mean set to zero was also performed on net transfer entropy for each condition to study cause-and-effect relationships. One-way ANOVA was used to compare the observables (time spent in the bottom of the tank, shoaling tendency, time spent freezing, transfer entropy from focal fish to replica, and transfer entropy from replica to focal fish) with the replica conditions as the independent variable. Upon significance of the one-way ANOVA, Tukey’s honest significant difference (HSD) post-hoc comparisons were conducted. To assess whether the appraisal of the robotic stimulus and the live counterpart by the zebrafish was comparable, we confronted the response of the focal fish in the condition 2-Fish with respect to all the replica conditions using a one-tail two-sample *t*-tests assuming equal variances of all the parameters. All the analyses were conducted with *p* < 0.05, except for the pairwise comparison, in which the statistical significance was determined based on a corrected *p*-value, which was set to 0.01 based on Bonferroni correction^75^.

### Data and materials availability

Datasets and codes used in the analyses are stored at the authors’ home institution and will be provided on request.

## Results

### Spatial preference and activity

**Figure 6:**
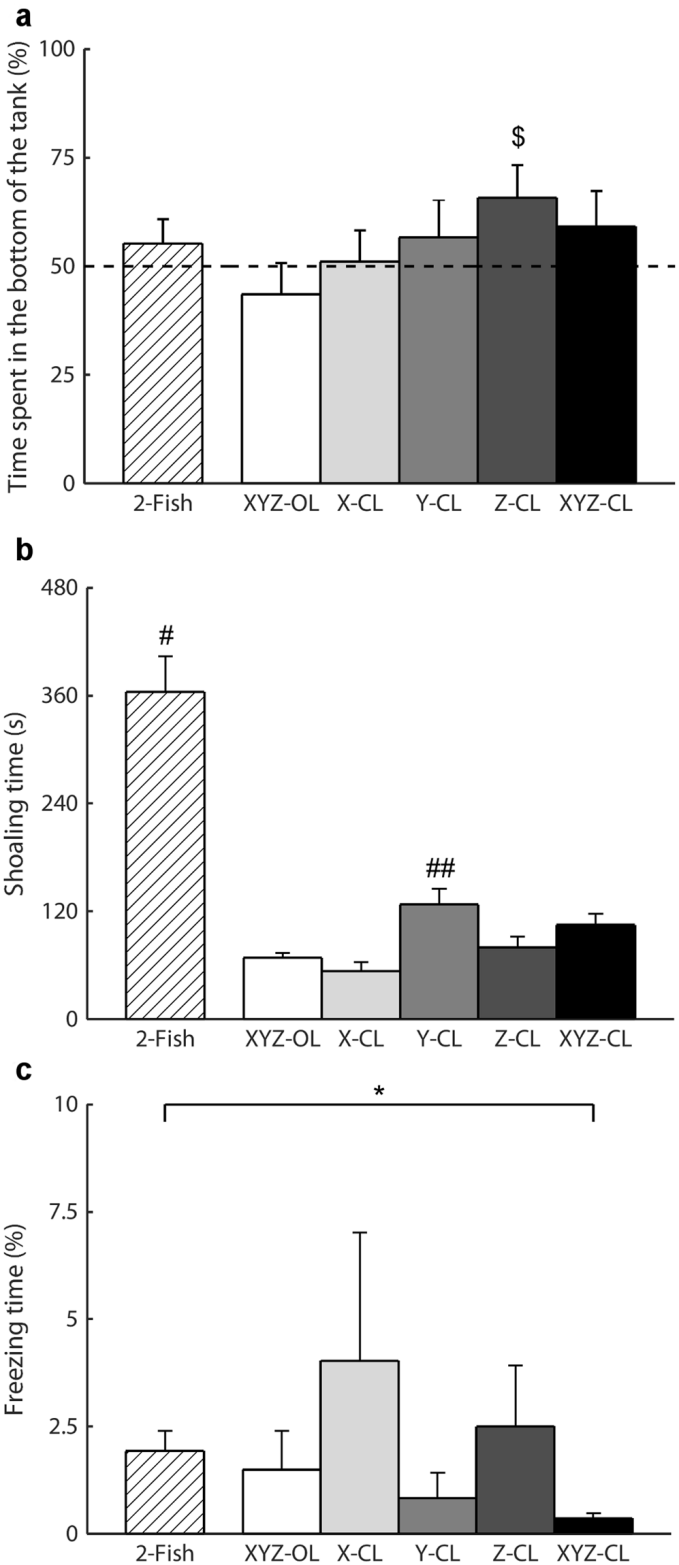
Analysis of spatial preference and activity. The bar plots show the time spent in the bottom of the tank (**a**), shoaling time (**b**), and time spent freezing by the focal fish (**c**) for the six experimental conditions (2-Fish: two live zebrafish; XYZ-OL: open-loop condition; X-CL: closed-loop with respect to the *X*-axis; Y-CL: closed-loop with respect to the *Y*-axis, Z-CL: closed-loop with respect to the *Z*-axis; and XYZ-CL: closed loop with respect to all axes). Dollar symbol indicates *p* < 0.05 in one-sample t-test comparison with chance, indicated as a dashed line. Octhorpe symbol indicates *p* < 0.01 in pairwise comparison with all other replica conditions. Double octhorpe symbol indicates *p* < 0.05 in post-hoc comparison with XYZ-OL and X-CL. Asterisk symbol indicates *p* < 0.01 in pairwise comparison. Data are represented as average + standard error.

Focal fish did not show preference for the bottom of the tank (2-Fish: *t*_9_ = 0.89, *p* = 0.1979; XYZ-OL: *t*_9_ = −0.85, *p* = 0.2095; X-CL: *t*_9_ = 0.14, *p* = 0.4451; Y-CL: *t*_9_ = 0.75, *p* = 0.2351; and XYZ-CL: *t*_9_ = 1.07, *p* = 0.1555), except for the Z-CL condition (*t*_9_ = 2.02, *p* = 0.0373) (Fig. 6a). One-way ANOVA did not reveal a difference in the preference of the focal fish for the bottom of the tank among the replica conditions (*F*_4,49_ = 1.08, *p* = 0.3788). Pairwise comparisons did not indicate a difference in the time spent in the bottom of the tank between the live stimulus condition, 2-Fish, and all the replica conditions (XYZ-OL: *t*_18_ = −1.21, *p* = 0.1201; X-CL: *t*_18_ = −0.43, *p* = 0.3345; Y-CL: *t*_18_ = 0.14, *p* = 0.4460; Z-CL: *t*_18_ = 1.08, *p* = 0.1472; and XYZ-CL: *t*_18_ = 0.38, *p* = 0.3534).

Shoaling time was found to vary across the replica conditions (*F*_4,49_ = 5.24, *p* = 0.0015), with condition Y-CL was significantly higher than in XYZ-OL and X-CL conditions (Fig. 6b). Pairwise comparisons indicated a difference in the shoaling tendency between the live stimulus condition, 2-Fish, and all the replica conditions (XYZ-OL: *t*_18_ = −6.96, *p* < 0.0001; X-CL: *t*_18_ = −7.21, *p* < 0.0001; Y-CL: *t*_18_ = −5.18, *p* < 0.0001; Z-CL: *t*_18_ = −6.52, *p* < 0.0001; and XYZ-CL: *t*_18_ = −5.93, *p* < 0.0001).

The analysis on the time spent freezing indicated no difference across the replica conditions (*F*_4,49_ = 0.79, *p* = 0.5350) (Fig. 6c). Pairwise comparison did not indicate a difference in the freezing time between 2-Fish and the replica conditions (XYZ-OL: *t*_18_ = −0.41, *p* = 0.3447; X-CL: *t*_18_ = 0.66, *p* = 0.2591; Y-CL: *t*_18_ = −1.39, *p* = 0.0905; and Z-CL: *t*_18_ = 0.36, *p* = 0.3617), except for the XYZ-CL condition (*t*_18_ = −3.09, *p* = 0.0032).

### Information transfer

**Figure 7:**
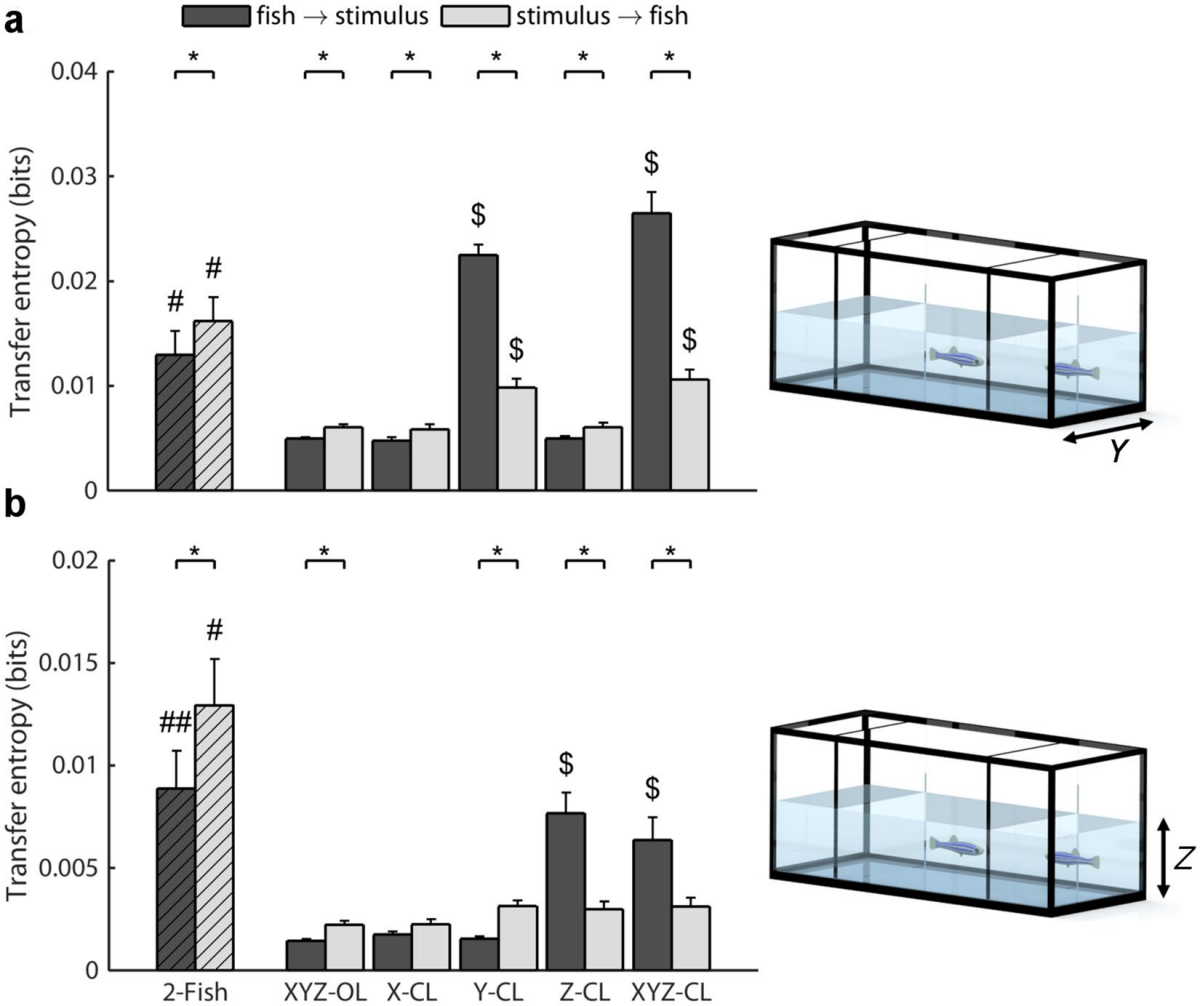
Analysis of the information transfer. The bar plots show the transfer entropy from focal fish to stimulus, and from stimulus to focal fish, along the *Y*-axis (**a**), and along the *Z*-axis (**b**) (2-Fish: two live zebrafish; XYZ-OL: open-loop condition; X-CL: closed-loop with respect to the *X*-axis; Y-CL: closed-loop with respect to the *Y*-axis, Z-CL: closed-loop with respect to the *Z*-axis; and XYZ-CL: closed loop with respect to all axes). Dollar symbol indicates significance in post-hoc comparisons (*p* < 0.05) with all the other replica conditions. Octothorpe symbol indicates a significant difference with respect to all the replica conditions at *p* < 0.01. Double octothorpes symbol indicates a significant difference with respect to conditions XYZ-OL, X-CL and Y-CL at *p* < 0.01. Asterisk identifies a significant difference in information transfer direction at *p* < 0.05. Data are represented as average + standard error.

Along the *Y-*axis, a net information flow from the stimulus to the focal fish was found in conditions: 2-Fish: *t*_9_ = −2.29, *p* = 0.0238; XYZ-OL: *t*_9_ = −2.72, *p* = 0.0118; X-CL: *t*_9_ = −1.89, *p* = 0.0460; Z-CL: *t*_9_ = −3.15, *p* = 0.0058; conversely, a net information flow from the focal fish to the stimulus was found in conditions: Y-CL: *t*_9_ = 7.12, *p* < 0.0001; and XYZ-CL: *t*_9_ = 7.85, *p* < 0.0001 (Fig. 7a). Transfer entropy from the focal fish to the stimulus was found to vary as a function of the condition (*F*_4,49_ = 73.47, *p* < 0.0001), with conditions Y-CL and XYZ-CL showing higher transfer entropy than conditions XYZ-OL, X-CL, and Z-CL. Similarly, the transfer entropy from the stimulus to the focal fish was found to vary as a function of the condition (*F*_4,49_ = 8.76, *p* < 0.0001), with conditions Y-CL and XYZ-CL showing higher transfer entropy than the conditions XYZ-OL, X-CL, and Z-CL.

Pairwise comparisons indicated a difference in the information transfer from the fish to the stimulus between 2-Fish condition and all the replica conditions (XYZ-OL: *t*_18_ = −3.32, *p* = 0.0019; X-CL: *t*_18_ = −3.37, *p* = 0.0017; Y-CL: *t*_18_ = 3.17, *p* = 0.0027; Z-CL: *t*_18_ = −3.30, *p* = 0.0020; and XYZ-CL: *t*_18_ = 4.21, *p* = 0.0003), while in the opposite direction, pairwise comparisons indicated a difference between 2-Fish condition and the replica conditions (XYZ-OL: *t*_18_ = −4.16, *p* = 0.0003; X-CL: *t*_18_ = −4.21, *p* = 0.0003; Z-CL: *t*_18_ = −4.14, *p* = 0.0003), except for the Y-CL and XYZ-CL conditions (Y-CL: *t*_18_ = −2.35, *p* = 0.0152; and XYZ-CL: *t*_18_ = −2.13, *p* = 0.0235).

Along the *Z-*axis, a net information flow from the stimulus to the focal fish was found in conditions: 2-Fish: *t*_9_ = −2.09, *p* = 0.0330; XYZ-OL: *t*_9_ = −2.84, *p* = 0.0097; Y-CL: *t*_9_ = −4.41, *p* = 0.0008; on the other hand, a net information flow from the focal fish to the stimulus was found in conditions: Z-CL: *t*_9_ = 4.07, *p* = 0.0014; and XYZ-CL: *t*_9_ = 3.33, *p* = 0.0044 (Fig. 7b). In X-CL condition, the analysis on the net transfer entropy failed to reach statistical significance (*t*_9_ = −1.62, *p* = 0.0697). Transfer entropy from the focal fish to the stimulus was found to vary as a function of the condition (*F*_4,49_ = 18.06, *p* < 0.0001), with conditions Z-CL and XYZ-CL showing higher transfer entropy than conditions XYZ-OL, X-CL, and Y-CL. On the other hand, transfer entropy from the stimulus to the focal fish did not vary as a function of the stimulus (*F*_1,19_ = 1.63, *p* = 0.1831).

Pairwise comparisons indicated a difference in the information transfer from the fish to the stimulus between 2-Fish condition and the replica conditions (XYZ-OL: *t*_18_ = −3.86, *p* = 0.0006; X-CL: *t*_18_ = −3.70, *p* = 0.0008; and Y-CL: *t*_18_ = −3.80, *p* = 0.0007), except for Z-CL and XYZ-CL (Z-CL: *t*_18_ = −0.55, *p* = 0.2930; and XYZ-CL: *t*_18_ = −1.12, *p* = 0.1381), while in the opposite direction, pairwise comparisons indicated a difference between the 2-Fish condition and all the replica conditions (XYZ-OL: *t*_18_ = −4.49, *p* = 0.0001; X-CL: *t*_18_ = −4.48, *p* = 0.0001; Y-CL: *t*_18_ = −4.07, *p* = 0.0004; Z-CL: *t*_18_ = −4.14, *p* = 0.0003; and XYZ-CL: *t*_18_ = −4.07, *p* = 0.0004).

## Discussion

In this study, we proposed a novel robotics-based platform to investigate the interactions between live zebrafish and biologically-inspired replica in a 3D workspace. A robotic arm was utilized to actuate the replica along four degrees of freedom, consisting of translations along three independent axes (two horizontal axes along the water surface and one vertical axis along the water column) and body oscillation. A custom-made real-time tracking software was developed to measure the position of a live zebrafish in the water tank, thereby affording the possibility of interactive experiments. Specifically, the real-time position of the live subject in 3D was used to independently control the motion of the replica along the three axes, such that the replica could follow the live subject during an experiment.

To quantify the role of the interactivity of the replica on the behavioural response of live subjects, we performed five experimental conditions, spanning from a full open-loop control, where the replica was actuated along predetermined trajectories replicating live stimuli, to closed-loop control along the three axes. Experiments were conducted in a canonical preference test, where focal subject swam in the central compartment of a water tank and the replica was actuated in a lateral compartment, separated by a transparent panel. Behavioural response was scored through the integration of traditional spatio-temporal measures, such as spatial preference and activity, and new information-theoretic quantities tailored to unravel cause-and-effect relationships underlying the interaction between fish and replica.

The results of this study indicated that spatial preference of zebrafish varied as a function of the control strategy used to actuate the replica. In post-hoc comparisons, we found that the shoaling time in the closed-loop control in the *Y*-axis (horizontal axis along the separating panels) was significantly higher than full open-loop control. The increased shoaling tendency could be viewed as an indicator of an improved degree of biomimicry of the replica actuated in closed-loop control in the *Y*-axis. In this condition, the replica was independently actuated along the *X*-axis (horizontal axis orthogonal to the separating panels) and *Z*-axis (vertical axis along the water column), mimicking pre-recorded trajectories of live stimuli, superimposed to a continuous following of the replica along the width of the experimental tank.

We may suggest that such a combination of closed- and open-loop control was the reason for the increased attraction of live subjects toward the replica with respect to full open-loop control. Zebrafish social interactions are highly based on visual cues, whereby individuals tend to quickly respond to swimming bouts of other conspecifics^76–78^. These swimming bouts belong to a well-documented, rich repertoire^52^; for example, agonistic or mating interactions entail instances of chasing, swimming in circles, and zig-zagging^63^. It is possible that the combination of closed- and open-loop control evoked a similar communication pattern between the replica and the focal fish, which translated into an increased shoaling tendency.

In partial disagreement with our intuition, increasing the degree of interactivity by implementing closed-control along the three axes did not translate into an improvement of the preference for the replica with respect to full open-loop control. This evidence could be related to the specific effect produced by closing the loop in the *X*-axis, which effectively positioned the replica in front of the separating panel. This might have led the focal fish to perceive that the replica was thrashing against the transparent wall. Thrashing could have been associated with an escape attempt of the replica^52^ or as an instance of aggression of the replica toward the live subject^63,79^. Both these behaviours could have triggered an avoidance response in the live subject, which was also indirectly evidenced by the reduction in shoaling tenency in closed-loop control in the *X*-axis with respect to closed-loop control in the *Y*-axis.

The dependence of the shoaling tendency on the degree of interactivity of the replica did not manifest into a variation of the time spent by the focal fish in the bottom of the tank. In all conditions, except of the condition in which we closed the loop only in the *Z*-axis, fish did not show a preference for the bottom of the tank. Geotaxis is a typical indicator of stress-related response in zebrafish, whereby diving toward the bottom of the tank is generally related to an avoidance response from a potential threat^52^. The lack of geotaxis accompanied by minimal freezing response suggest that the platform was unlikely perceived as a potential threat by the focal subjects. This is different from our previous experiments^53^, where we found a consistent geotactic response, which prompted us to reconsider the experimental apparatus using black fabric curtains to conceal the robotic platform. These curtains might have prevented the focal fish from perceiving the hardware extending over the tank as an aerial predator, which could elicit diving response.

The preference for the bottom of the tank registered when closing the loop in the *Z*-axis could be related to the continuous following movements of the replica along the water column, which might have been perceived by the focal fish as a signal of alarm response. Specifically, it is possible that tracking errors in the real-time tracking software resulted in unwanted, sudden movements of the replica along the water column. These movements could have elicited a stress-related response in the subjects, in the form of increasing diving^52,63^. However, current data challenges the precise quantification of the relationship between tracking errors and increased diving. Future experiments might seek to detail zebrafish alarm responses to the replica, by systematically controlling the extent and frequency of sudden movements along the water column. Alternatively, we could hypothesize that zebrafish spent more time in the bottom of the tank in an attempt to gain an optimal view of the interactive swimming bouts of the replica along the water column. This hypothesis rests upon recent findings on the visual acuity of zebrafish^80^, which suggest that zebrafish may perceive and therefore preferentially react to conspecifics swimming above them. In this case, time spent in the bottom of the tank would be interpreted as a form of inspection with the replica, rather than a stress-related response.

Building on our previous work^53,55,59,65^, we used transfer entropy to infer influences from the motion of the live fish and replica. Our results confirmed the hypothesis that information flow between the replica and the live fish was regulated by the selection of the control strategy. In agreement with our expectations, we found that closing the loop only in the *Y-* or *Z-*axis resulted into a differential information flow between the replica and the live fish with respect to the motions along the two axes. Specifically, closing the loop in the *Y-*axis (*Z*-axis) resulted in the live fish influencing the motion of the replica along the same axis, whereby the actuation of the replica along that axis was controlled in real-time by the motion of the focal subject. At the same time, closing the loop in the *Y-*axis (*Z*-axis) yielded to the replica influencing the motion of the live fish along the *Z-*axis (*Y*-axis), for which open-loop control was implemented and the live fish was responsive to the replica. Predictably, closing the loop in all the axes resulted in the live fish influencing the motion of replica in both the *Y-* and *Z-*axis. We also extended our previous claims on open-loop^53,65^ control to 3D, whereby we found influence of the replica on the live subject with respect to both the *Y-* and *Z-*axis.

From the analysis of transfer entropy, we also recorded a dependence of the strength of the information flow on the degree of interactivity of the condition. Specifically, closing the loop in the *Y*-axis resulted into an increase in information flow with respect to the motion along the same axis, which should be considered as an indication of an improved interaction between the live subject and the replica. Not only did the influence of the live fish on the replica increased due to closed-loop control, but also the influence of the replica on the live fish increased due to the positive feedback produced by interactivity. A similar trend was registered from the closed-loop control in the *Z*-axis, although we failed to observe an increase in the influence of the replica on the live fish with respect to the motion along the same axis in open-loop control.

The major technical advancements of this robotic platform, with respect to our previous work^53^ resides in improvements to the hardware and software systems. Hardware improvements included the addition of the stepper motor for controlling heading angle, the attachment of the transparent pulley system to this stepper motor, and the concealment of the mechanisms of the robotic platform with the black fabric curtains. These changes sought to potentially afford continuous rotation of the heading of the replica and reduce the perception of the robotic platform actuation as an aerial predator.

The software enhancements of the robotic platform consisted of the ease of implementation of control strategies, real-time data acquisition, processing speed, and motor control. Specifically, the control system uses the same setup as the open-loop robotic platform as our previous work^53^, and hardware changes were not needed to achieve closed-control. Further, the capability to simultaneously acquire and save the position data of the stimulus and focal fish in real-time reduced the effort needed to track and analyse the recorded video in post-processing. Additionally, the real-time tracking software operated at 30 Hz, which was on the faster end of the spectrum in comparison to other studies with a real-time tracking software (ranging between 0.1 to 30 Hz)^31,32,34,37,40,43^. The use of the master microcontroller to incrementally ramp the voltage delivered to the DC motor aided in reducing jitteriness that arose when the robotic platform was traveling on the *Y*-axis.

Although the new robotic platform contributed a number of hardware and software advancements for the implementation of biomimetic robotic stimuli, the larger shoaling tendency of zebrafish toward live conspecifics suggest that the replica was not perceived as conspecifics in any condition. Based on our recent findings^54^, we could have expected closed-loop control to allow for an equivalent appraisal of the robotic and the live stimuli. Specifically, we showed that zebrafish exhibit an equivalent level of preference for replicas controlled in open-loop and live conspecifics separated by one-way glasses that did not allow for social feedback^54^. Grounded in this evidence, we could have anticipated that focal subjects would display an equivalent spatial preference for live stimuli separated by a transparent panel and an interactive replica, controlled in real-time to follow the focal subject. A number of factors might have contributed to the reduced attraction for the interactive replica with respect to a live stimulus, including mechanical limitations of the platform and limited degree of biomimicry of the interactive control.

With respect to mechanical limitations, we identified three specific issues that could have hindered the attractive value of the robotic stimulus. First, since the trajectory of the focal fish was scaled down to fit the workspace of the platform and prevent the collision of the replica to the wall, the replica was consistently slower than the live fish (see supplementary material). A second issue was the potential slippage at the casing holding the DC motor (about 1 mm clockwise or counter-clockwise with a frequency of few occurrences per minute), which might not have been compensated by the encoder, thereby distorting the motion along the *Y*-axis. Finally, the oscillatory motion of the replica was only modulated by the translation along the *Y*-axis, which could have resulted in instances of unnatural motion, with the replica potentially moving backwards (few times per trial for short bouts of approximately one second in duration). Future work should seek to improve on the platform to afford more fluid and faster motions of the replica, possibly integrating a dedicated control system for trajectory tracking by the robotic arm.

The interactive control was also subject to practical limitations in: (i) the reaction time, which was on the order of 0.5 s; (ii) target accuracy, which could have resulted in false or distorted commands to the platform; and (iii) the implemented strategy, which forced the replica to merely follow the subject. With respect to the first limitation, we must acknowledge that the reaction time in live fish is as fast as 20 ms, as seen in startle responses of several teleost fish (including zebrafish)^81^. A number of factors were likely to affect the target accuracy, including uncertainties in the 2D measurement of fish trajectories and their 3D interpolation, along with imprecise appraisal of the workspace.

The third limitation warrants a more detailed examination. In fact, the interaction between conspecifics is highly nonlinear and is composed of a complex collection of visual cues and sophisticated swimming patterns^63,76,77^. For example, Oliveira *et al*.,^82^ studied zebrafish behavioural phenotypes during an agonistic interaction between dyads of males. They found that two fish may frontally approach each other in a mutual assessment, which includes behaviours like displays, circling, and biting. After the resolution of the aggression instance, the winning zebrafish starts chasing the subordinate, which flees or freezes in return. Another example is the study of zebrafish mating by Darrow and Harris^83^, where they observed how females systematically respond with a specific behaviour to the complex courtship display of fish of the opposite sex. The premise of the selected control scheme in which the replica would follow specific movements of the live fish was to offer a first demonstration of 3D interactivity in robotics-based experiments on zebrafish. The limited complexity of the interactive scheme was also evidenced in the comparison with the information flow between conspecifics, which revealed a richer interaction than those attained through our robotics-based platform. Future work should explore alternative control strategies to enhance the degree of biomimicry of interacting replica.

In conclusion, this study demonstrated, for the first time, an interactive robotics-based approach for zebrafish behavioural phenotyping in 3D. The platform is expected to find application in a number of translational studies involving this powerful animal model, such as the investigation of the pathogenesis of brain disorders and their underlying molecular and neurogenetics mechanisms. Several studies have already acknowledged the potential of a robotics-based approach to zebrafish research toward new treatments for neurological diseases^60,62^ and an improved understanding of anxiety and cognitive deficits^61^. In these efforts, there is a critical need for interactive technologies that could help elucidating functional and dysfunctional processes in the zebrafish animal model.

## Electronic supplementary material

Supplementary material: Closed-loop control of zebrafish behavior in three dimensions using a robotic stimulus

Video S1. Front/top sample video of one experiment of open loop condition

Video S2. Front/top sample video of one experiment of closed loop condition.

Video S3. Front/top sample video of one experiment of fish-fish condition.
