# Supplementary material for: Closed-loop control of zebrafish behaviour in three dimensions using a robotic stimulus
Source: Sci Rep. 2018 Jan 12;8:657. doi: 10.1038/s41598-017-19083-2 (PMC5766612; doi:10.1038/s41598-017-19083-2)
Supplement: Supplementary file 1 — Supplementary material: Closed-loop control of zebrafish behavior in three dimensions using a robotic stimulus [file 41598_2017_19083_MOESM1_ESM.pdf]

Supplementary material: Closed-loop control of zebrafish behavior in three dimensions using a robotic stimulus

Changsu Kim, Tommaso Ruberto, Paul Phamduy, and Maurizio Porfiri*

Department of Mechanical and Aerospace Engineering, New York University Tandon School of Engineering, Brooklyn, New York, 11201, USA

*To whom correspondence should be addressed: mporfiri@nyu.edu, +1 (646) 997-3681 (phone), +1 (646) 997-3532 (fax)

Control of the robotic platform motors

The master microcontroller was programmed to incrementally ramp the voltage delivered to the DC motor at ±6.1 V/s toward the desired position along the *Y*-axis, within the limits of -12 and 12 V. Based on technical specifications of the motors and rack-and-pinion dimensions, the maximum speed of the platform along the *Y*-axis was estimated to be 10.4 cm/s. The master microcontroller also controlled the heading angle of the replica. From a bird's eye view, the heading angle was incrementally ramped at 86°/s counterclockwise when the replica was moving along the *Y*-axis direction, and clockwise when the motion was in the opposite direction. The heading of the replica has a range of 180°, with the 0° facing toward the center compartment along the *X*-axis and ±90° identifying the direction of the *Y*-axis. The same microcontroller was used for the motion along the *X*-axis at a maximum speed of 5.8 cm/s, while the slave microcontroller was used for the motion along the *Z*-axis at a maximum speed of 5.1 cm/s.

Interpolation process to infer 3D coordinates

A critical element in our real-time implementation was to compensate for the distortion associated with the perspective view from each camera. In the description of our interpolation process, all coordinates are referenced to the origin located at the center of the water tank from the top view and at the height of the water level in the front view.

From the tracking software, we obtained the 2D coordinates of the target (X_{2D}, Y_{2D}, Z_{2D}) as functions of time, where the *X*- and *Y*- coordinates were taken from the top view, and the *Z*- coordinate was taken from the front view. The *X*- coordinate from the front view was disregarded. The 2D coordinates, measured in centimeters, are denoted with a subscript "2D" to emphasize their derivation from the independent 2D views.

Before each experimental trial, we performed a simple calibration using a single frame from the two cameras, and the pixel position of the corners of the near, and far side walls of the water tank (relatively to the front camera) were manually extracted from the frames. The length of the tank, width of the tank, and height of the water level (half of the height of the tank) were measured in pixels for both perspectives using the two frames (see figure S1). The length of the tank, width of the tank, and height of the water level inferred from the near side perspective are labelled as l_X^n, l_Y^n, and l_Z^n , and these same quantities from the far side perspective are termed l_X^f, l_Y^f, and l_Z^f, respectively. Measured values for the near and far side perspective lengths are tabulated in table S2.

These quantities were confronted with the physical dimensions of the swimming tank for calibration – we use the notation l_X, l_Y, and l_Z for the length of the tank, the width of the tank, and the water level, which are 74, 30, and 15 cm, respectively. The coordinates of the target obtained after interpolation are denoted with a subscript "3D" (X_{3D}, Y_{3D}, and Z_{3D}) and were measured in centimeters.

Briefly, the process of interpolation consisted of the following steps. First, we interpolated the 2D *Z*- coordinate of the target (Z_{2D}) in the front view. The Y_{2D}-coordinate from the top view (ranging from $-l_Y/2$ to $l_Y/2$) was utilized to determine the value of the interpolated *Z*-coordinate between the far and near perspectives, based on Z_{2D} in the front view. The Z_{3D}-coordinate was obtained by scaling Z_{2D} by factor between l_Z^n/l_Z^f and 1 corresponding to the far and near perspective, respectively (equation 1).

$$Z_{3D} = Z_{2D} \frac{l_Z^n}{\frac{\left(Y_{2D} + \frac{l_Y}{2}\right)}{l_Y}(l_Z^n - l_Z^f) + l_Z^f} \tag{1}$$

After resolving the *Z*-coordinate of the focal fish, Z_{3D}, we interpolated the *X*- and *Y*-coordinates between the far and near perspective, based on the X_{2D} and Y_{2D} on the top view. Specifically, X_{2D} was scaled by a term ranging between 1 and l_X^n/l_X^f, corresponding to the near and far perspective, respectively. Similarly, Y_{2D} was scaled by a term ranging between 1 and l_Y^n/l_Y^f, corresponding to the near and far perspective, respectively. The *X*- and *Y*-coordinates of the target after interpolation are shown equation 2 and 3.

$$X_{3D} = X_{2D} \frac{l_X^n}{\frac{Z_{3D}}{l_Z}(l_X^f - l_X^n) + l_X^n} \tag{2}$$

$$Y_{3D} = Y_{2D} \frac{l_Y^n}{\frac{Z_{3D}}{l_Z}(l_Y^f - l_Y^n) + l_Y^n} \tag{3}$$

Analysis on average speed of focal fish and stimuli

The speed of the focal fish and stimuli were computed from the distance traveled between consecutive frames and averaged across all trials. One-way ANOVA was used to compare the average speed of focal fish and stimuli with the replica conditions as the independent variable. To assess whether the appraisal of the robotic stimulus and the live counterpart by the zebrafish was comparable, we confronted the average speed of the focal fish and of the stimulus in the condition 2-Fish with respect to all the replica conditions using a one-tail two-sample *t*-tests assuming equal variances. All the analyses were conducted with $p<0.05$, except for the pairwise comparison, in which the statistical significance was determined based on a corrected *p*-value, which was set to 0.01 based on Bonferroni correction (*1*).

The average speed of the focal fish was found not to vary across the replica conditions ($F_{4,49} = 0.88, p = 0.4846$) (figure S2). Pairwise comparisons did not indicate a difference in the focal fish average speed between the 2-Fish and the replica conditions (XYZ-OL: $t_{18} = 1.88, p = 0.0382$; X-CL: $t_{18} = 0.21, p = 0.4196$; Y-CL: $t_{18} = 2.16, p = 0.0223$; and Z-CL: $t_{18} = 1.17, p = 0.1282$), except for the XYZ-CL condition ($t_{18} = 2.63, p = 0.0084$).

The average speed of the stimulus was not found to vary across the replica conditions ($F_{4,49} = 0.32, p = 0.8603$) (figure S3). Pairwise comparisons did not indicate a difference in the stimulus average speed between condition 2-Fish and the replica conditions (XYZ-OL: $t_{18} = 2.24, p = 0.0190$; Z-CL: $t_{18} = 2.31, p = 0.0164$; and XYZ-CL: $t_{18} = 2.54, p = 0.0103$), except for X-CL ($t_{18} = 2.76, p = 0.0065$), and Y-CL ($t_{18} = 2.61, p = 0.0088$).

The increase of the average speed in closed-loop control along the three axes may be related to the specific effect produced by closing the loop in the *X*-axis. As explained in the

manuscript, this might have led the focal fish to perceive that the replica was thrashing against the transparent wall, thereby triggering an escape response in the focal subjects.

Our findings indicate that the replica was generally slower than the live stimulus, with a significant difference attained when closing the loop in the *X*- or *Y*-axis. This decrease could be explained by the reduced workspace of the replica with respect to the compartment, which might have resulted in a reduced average speed of the replica.

References

1. G. Rupert Jr, *Simultaneous statistical inference*. (Springer Science & Business Media, New York, NY, USA, 2012).

Figures and tables

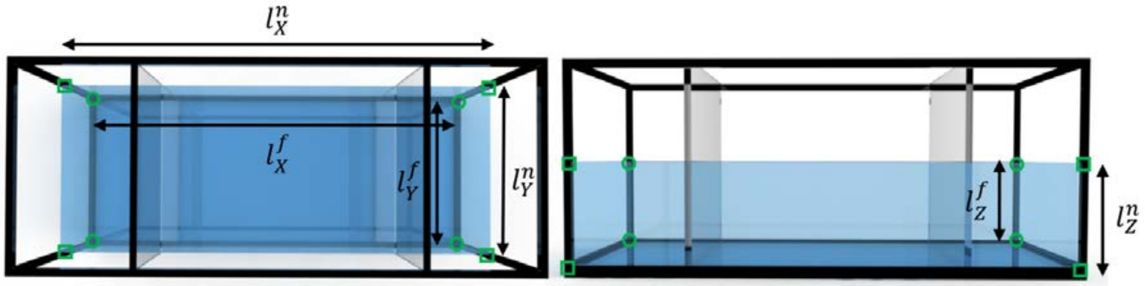

Figure S1. Perspectives from the top and front views. The drawings show the dimensions of the water tank from the top (left) and front (right) views.

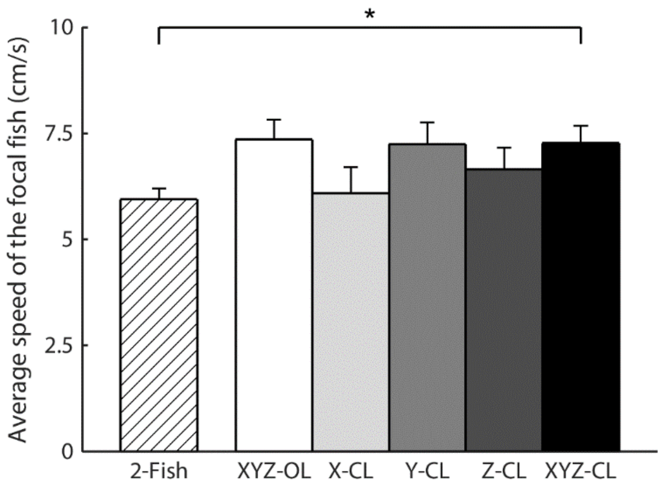

Figure S2. Speed of the focal fish. The bar plot shows the average speed of the focal fish for the six experimental conditions (2-Fish: two live zebrafish; XYZ-OL: open-loop condition; X-CL: closed-loop with respect to the X axis; Y-CL: closed-loop with respect to the Y-axis, Z-CL: closed-loop with respect to the Z-axis; and XYZ-CL: closed loop with respect to all axes). The asterisk symbol indicates $p < 0.01$ in pairwise comparison with XYZ-CL. Data are represented as average + standard error.

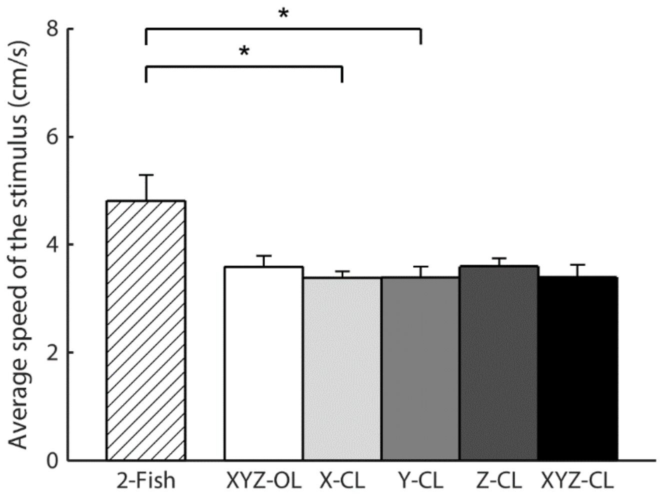

Figure S3. Speed of the stimuli. The bar plot shows the average speed of the stimulus for the six experimental conditions (2-Fish: two live zebrafish; XYZ-OL: open-loop condition; X-CL: closed-loop with respect to the X axis; Y-CL: closed-loop with respect to the Y-axis, Z-CL: closed-loop with respect to the Z-axis; and XYZ-CL: closed loop with respect to all axes). The asterisk symbol indicates $p < 0.01$ in pairwise comparison with X-CL and Y-CL. Data are represented as average + standard error.

Table S1. Interpolation symbols. The table synoptically defines the symbols utilized in the interpolation of the 3D coordinates of the focal fish and replica.

Description	Label	Units
near side perspective lengths	l_X^n, l_Y^n, l_Z^n	pixel
far side perspective lengths	l_X^f, l_Y^f, l_Z^f	pixel
physical dimensions	l_X, l_Y, l_Z	cm
raw 2D target coordinates	X_{2D}, Y_{2D}, Z_{2D}	cm
interpolated 3D target coordinates	X_{3D}, Y_{3D}, Z_{3D}	cm

Table S2. Measured values for the near and far side perspective lengths. The table reports the average and standard deviations of near and far side perspective lengths in pixels, computed based on the entire set of 60 trials considered in this study.

Perspective	Dimension	Label	Average (pixels)	Standard deviation
near side	length of the tank	l_X^n	498.5	11.5
near side	width of the tank	l_Y^n	202.1	10.0
near side	height of the water level	l_Z^n	121.2	4.7
far side	length of the tank	l_X^f	448.8	4.8
far side	width of the tank	l_Y^f	177.9	21.6
far side	height of the water level	l_Z^f	90.2	5.0

Video S1. Front/top sample video of one experiment of open loop condition. The sample video shows a portion of a trial for the open loop (OL) condition.

Video S2. Front/top sample video of one experiment of closed loop condition. The sample video shows a portion of a trial for the closed loop (CL) condition.

Video S3. Front/top sample video of one experiment of fish-fish condition. The sample video shows a portion of a trial for the fish-fish (2-Fish) condition.
